# Data Integration Workflow for Search of Disease Driving Genes and Genetic Variants

**DOI:** 10.1371/journal.pone.0018636

**Published:** 2011-04-12

**Authors:** Sirkku Karinen, Tuomas Heikkinen, Heli Nevanlinna, Sampsa Hautaniemi

**Affiliations:** 1 Sirkku Karinen Institute of Biomedicine and Genome-Scale Biology Research Program, University of Helsinki, Helsinki, Finland; 2 Tuomas Heikkinen Department of Obstetrics and Gynecology, Helsinki University Central Hospital, Helsinki, Finland; 3 Heli Nevanlinna Department of Obstetrics and Gynecology, Helsinki University Central Hospital, Helsinki, Finland; 4 Sampsa Hautaniemi Institute of Biomedicine and Genome-Scale Biology Research Program, University of Helsinki, Helsinki, Finland; Ohio State University Medical Center, United States of America

## Abstract

Comprehensive characterization of a gene's impact on phenotypes requires knowledge of the context of the gene. To address this issue we introduce a systematic data integration method Candidate Genes and SNPs (CANGES) that links SNP and linkage disequilibrium data to pathway- and protein-protein interaction information. It can be used as a knowledge discovery tool for the search of disease associated causative variants from genome-wide studies as well as to generate new hypotheses on synergistically functioning genes. We demonstrate the utility of CANGES by integrating pathway and protein-protein interaction data to identify putative functional variants for *(i)* the *p53* gene and *(ii)* three glioblastoma multiforme (GBM) associated risk genes. For the GBM case, we further integrate the CANGES results with clinical and genome-wide data for 209 GBM patients and identify genes having effects on GBM patient survival. Our results show that selecting a focused set of genes can result in information beyond the traditional genome-wide association approaches. Taken together, holistic approach to identify possible interacting genes and SNPs with CANGES provides a means to rapidly identify networks for any set of genes and generate novel hypotheses. CANGES is available in http://csbi.ltdk.helsinki.fi/CANGES/

## Introduction

Cellular functions are regulated by complex and multivariate molecular regulatory networks. Complex diseases, such as cancers, arise from alterations in these networks and thus contribution of any single gene to disease risk or progression should be viewed in the context of molecular networks [1], [2]. Indeed, genome-wide measurement technologies, such as gene and single nucleotide polymorphism (SNP) microarrays, have provided an opportunity to identify genes that are mutated or differentially expressed and drive various diseases. In particular, SNP-arrays have been powerful in genome-wide association (GWA) studies and have resulted in several genetic loci or genes that are associated with disease risk or poor prognosis [3]–[5]. As such *candidate genes* typically affect cellular functions by altering signaling in regulatory networks, it is crucial to comprehensively characterize these regulatory networks.

We introduce a data integration workflow CANGES (Candidate Genes and SNPs) to rapidly identify *focal genes*, *i.e.*, genes that code for proteins which interact or belong to the same molecular network with a protein coded by a candidate gene. Additionally, CANGES is able to identify *central SNPs*, *i.e.*, genetic variations that are located in the focal genes' coding or regulatory regions, such as splice and 3′ UTR sites (Table S1). Thus, central SNPs may affect protein function and cause gene-gene and SNP-SNP interactions in the regulatory network leading to increased risk or survival effects.

To identify focal genes, CANGES uses KEGG and Reactome pathway databases [6], [7] together with PINA protein-protein interaction (PPI) database [8]. The focal genes are further queried for their SNPs using ENSEMBL [9] and linkage disequilibrium information from HapMap [10]. The impacts of non-synonymous coding SNPs to protein functions are then predicted using PolyPhen [11], PolyPhen-2 [12], SNPs3D [13] and SIFT [14]. Accordingly, CANGES can rapidly identify genes and SNPs belonging to the same network of any set of genes.

CANGES integrates data from SNP, protein and linkage disequilibrium databases and thus belongs to the meta-server class of services [15]. Earlier meta-approaches have focused on predicting SNP functions and linking them to GWA studies [16]–[18], enriching SNPs with pathway- and functional annotations [16], [17], linking SNP annotations with PPI information [19], collecting functional predictions for SNPs from a number of sources for one SNP at a time [20] or collect and rank central SNPs for specified genes and ontologies [21]. A unique feature in CANGES is that, instead of just annotating genes, it takes an advantage of the accumulated pathway and PPI information to provide a comprehensive list of focal genes and central SNPs with one batch query. For the more conventional use, CANGES enables focal gene search based on the pathway name and SNP retrieval for a custom gene list. CANGES also provides a means to link central SNPs to tag-SNPs, which facilitates search for putative disease causing alleles and enables integration of data from several SNP-arrays that typically use different sets of tag-SNPs. Each CANGES query produces a downloadable result list, which enables easy processing and comparison of the output. CANGES runs on a freely available component-based bioinformatics workflow environment Anduril [22], which allows the use of CANGES as a part of re-usable analysis workflows. Additionally, we have created a web interface for smaller queries.

## Results

**Figure 1:**
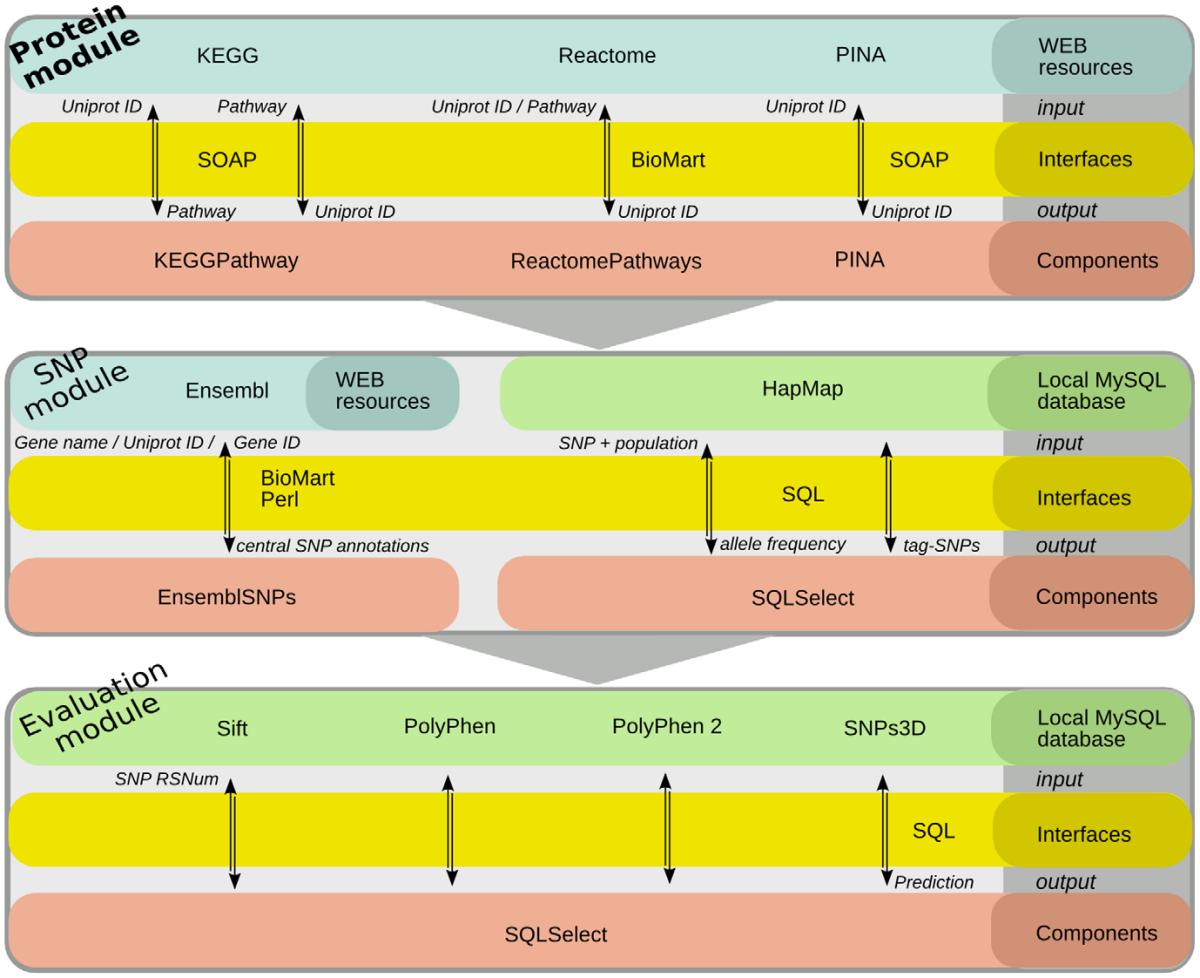
Workflow of CANGES modules and components, resources and interfaces within each module. The input of the protein module is a list of protein identifiers or pathway names. The output is a list of corresponding focal genes, *i.e.*, genes that belong to the same pathways with query protein or query pathway in KEGG or Reactome, or whose protein products interact with the query proteins. The resulted focal genes are inputs for searching the central SNPs (SNP module) using ENSEMBL and HapMap. The central SNPs are first queried from ENSEMBL and then the allele frequencies and tag-SNPs are fetched from a local HapMap database. The SNP module returns a list of central SNPs with their annotations and tag-SNPs. The list of central SNPs is then passed for the evaluation module in which the SNPs are then further evaluated with four methods that predict the functional effects of SNP to protein function.

CANGES workflow diagram, including modules, resources, bioinformatics components and interfaces, is shown in Figure 1. CANGES produces the results as Excel spreadsheets that enable an easy sorting and filtering of the results. An example of an CANGES produced result file is given in Table S2. Here, we demonstrate the utility of CANGES with two case studies. The first case study illustrates CANGES analysis for a single candidate gene *p53* and the second one focuses on assessing the context for three genes reported in a recent glioblastoma GWA study.

### Comprehensive catalogue of SNPs for the *p53* associated pathways and interactome

**Figure 2:**
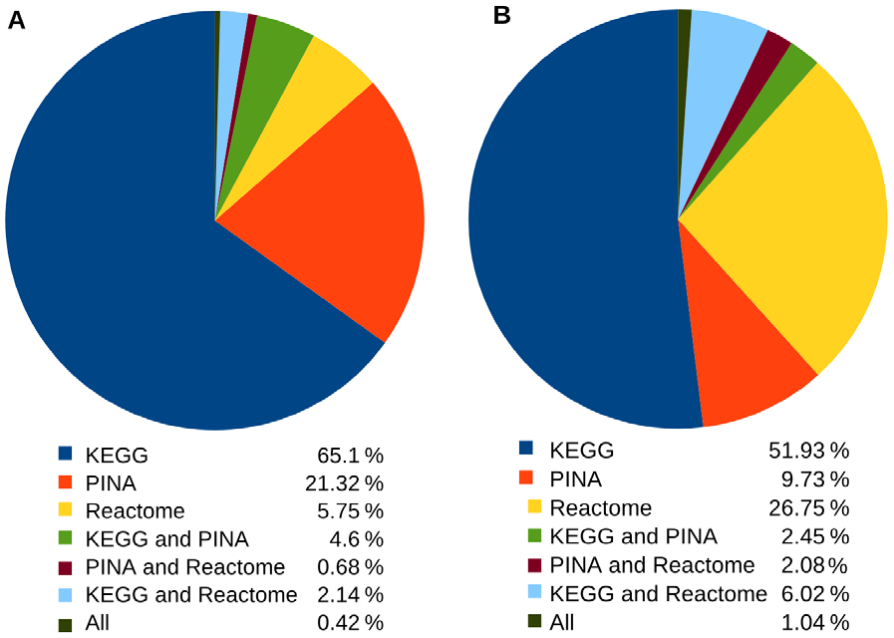
Distribution of the database sources. The database sources of focal genes for a) *p53* and b) glioblastoma multiforme case study.

The gene *p53* is one of the most studied genes in cancer biology. However, the function and interplay of *p53* with the other proteins is still unclear. We used CANGES to provide the most comprehensive set of proteins that function within the same pathways with *p53* or have direct PPI with *p53* [Swiss-Prot:P04637, Q9NP68] (Table S2). CANGES analysis produced 1,914 focal genes for *p53* that were identified from databases as illustrated in Figure 2A). Given the importance of *p53* mutations in cancer susceptibility and progression, we further queried central SNPs for the *p53* focal genes. This search resulted in 47,163 central SNPs and 3,465 tag-SNPs that tag 1,720 of the central SNPs. The threshold for selecting the tag-SNPs was 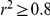.

To further identify SNPs that putatively have functional effect at the protein level, we analyzed the 47,163 central SNPs with four methods. PolyPhen and PolyPhen-2 predicted 1,479 and 672 SNPs as functional with probable or possible damaging effect on protein, respectively. SNPs3D predicted 2,000 SNPs being functional, whereas SIFT resulted in 2,435 functional SNPs. These prediction methods agreed for 158 SNPs (Table S3). Genes associated with the set of 158 SNPs with predicted functional effects at the protein level consists of several genes that are crucial in pathogenesis, such as *ATM*, *CDKN2A*, *BRCA1* and *BRCA2*. To our knowledge, this catalogue is the most comprehensive list of SNPs putatively affecting *p53* mediated signaling.

### Identification of survival associated SNPs in glioblastoma multiforme

Glioblastoma multiforme (GBM) is grade IV glial originating tumor type that responds poorly to all available therapeutic regimens and has a median survival of one year [3]. In a recent glioma GWA study Shete and colleagues identified low-penetrance susceptibility loci harboring *TERT* (Swiss-Prot: O14746, Q9UNR4, Q8NG38, Q9UBR6, Q9UNS6), *CCDC26* (Swiss-Prot: Q8TAB7), *CDKN2A* (Swiss-Prot: Q8N726, P42771, Q208B5, Q5ZEY9, Q9UPB7, A7LNE7, A5X2G7, Q2MJK0), *CDKN2B* (Swiss-Prot: P42772, Q5ZEY8, O15125, Q8NIA6, Q9UM95), *RTEL1* (Swiss-Prot: Q9BW37 Q9NZ71) and *PHLDB1* (Swiss-Prot: Q86UU1, B0YJ63, B0YJ65) with elevated risk of glioma [5]. We queried focal genes using these six glioma-associated genes. *TERT*, *CDKN2B* and *CDKN2A* were found in KEGG, Reactome or PINA. These three genes resulted in a total of 1,346 focal genes (Figure 2B), which were annotated for their central SNPs. This resulted in 33,428 central SNPs, for which we found 2,657 tag-SNPs having pair-wise correlation with a central SNP 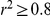; 463 SNPs were both central and tag-SNPs totaling to 35,622 SNPs for further analysis (Table S4).

We used The Cancer Genome Atlas (TCGA) GBM data set of 209 samples subjected to 550k SNP-array experiments to identify SNPs associated with poor survival using Kaplan-Meier analysis with log-rank test. We initially considered all 35,622 central and tag-SNPs for the survival analysis. However, only 1,888 SNPs were found from the TCGA genome-wide SNP-array for GBM. This small number is due to the low number of central SNPs in the SNP-arrays in general; here, 0.02% of the central SNPs were on the array. We were able to estimate the survival effect for 995 central SNPs directly or through their tag-SNPs. Survival analysis of the set of 1,888 SNPs resulted in 18 SNPs with a significant survival effect. From these, eight were central SNPs, and 10 SNPs further tag 18 central SNPs totaling to 26 central SNPs putatively having survival impact.

**Figure 3:**
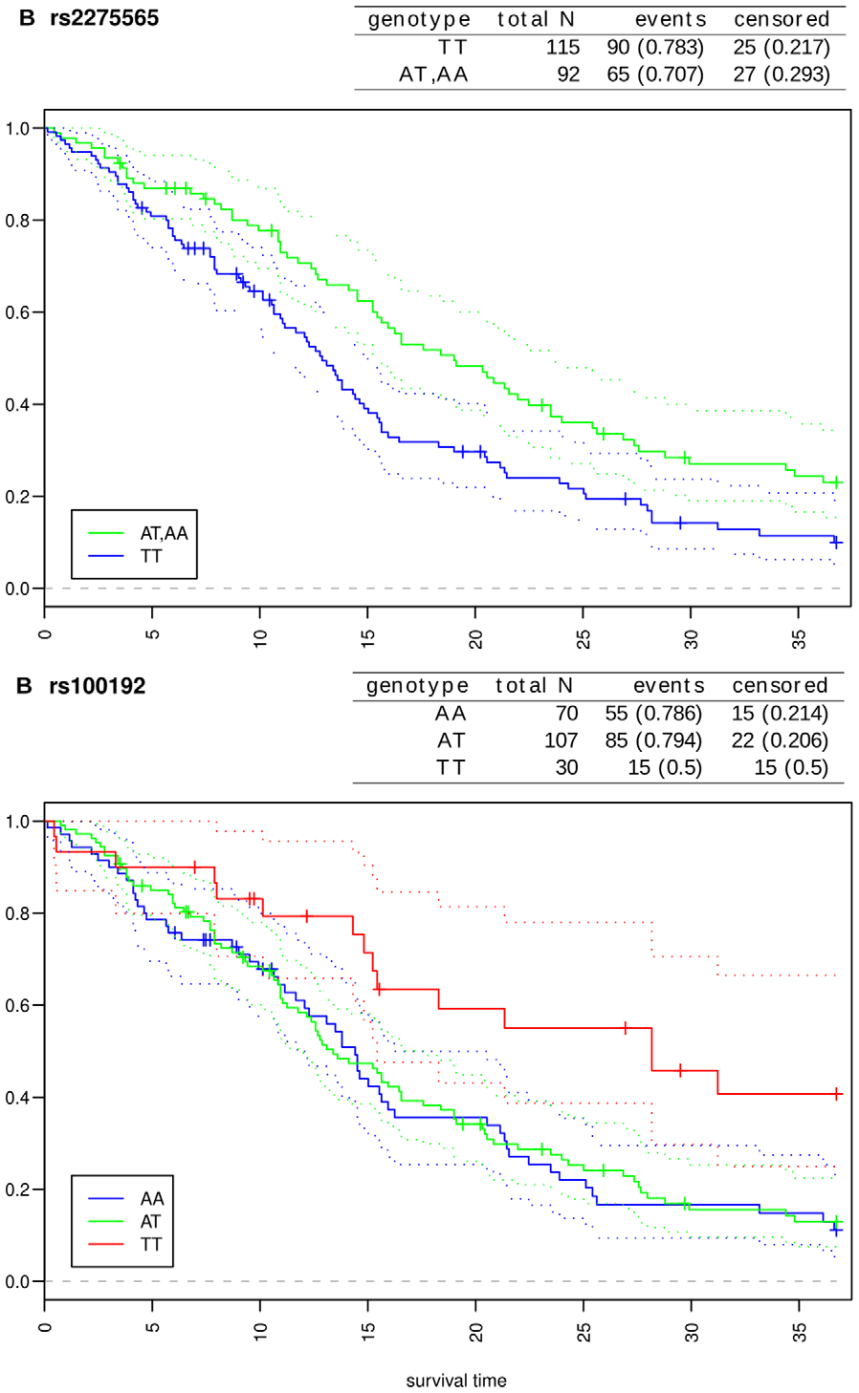
Kaplan-Meier estimates and the number of cases of the genotype groups for the GBM survival analysis. Dashed lines illustrate 0.95 confidence intervals for each group in the analysis. Panel a) rs2275565 is a tag-SNP to the coding SNP rs1805087 in the *MTR* gene. The group of 11 homozygous cases with AA genotypes is combined with the heterozygous group. Panel b) rs100192 is a tag-SNP to a SNP rs164390 in 5′ UTR of the *CCNB1* gene.

We then mapped the set of 26 central SNPs with putative survival effect to genes. The SNPs are located in 14 genes: *CAMK2D*, *CCNB1*, *CD82*, *CEP192*, *CLASP2*, *FLT3*, *KIF2B*, *LAMA1*, *MTR*, *PML*, *PSMF1*, *SEC13*, *SGOL2* and *SMAD5*. The lowest p-value (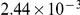) was observed for the tag-SNP rs2275565 in the intronic region of the *MTR* gene. In addition, the most significant odds ratio between homozygous genotypes was calculated for the tag-SNP rs100192 (odds ratio 0.27 with p-value 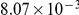) in the intronic region of the *CENPH* gene. This SNP tags rs164390 in 5′ UTR region of a gene G2/mitotic-specific cyclin-B1 (*CCNB1*). Kaplan-Meier estimates for rs2275565 and rs100192 are illustrated in Figure 3, and the other figures with CANGES output table are available in Figure S1 and Table S5.

## Discussion

A number of individual cancer associated genetic variants have been found recently, but the signaling-level context for these variants has been challenging to establish. Finding genetic variants causing functional effects to a protein network, which also contains a risk or cancer-driving gene's protein product, are of particular interest as these are candidates for SNP-SNP or gene-gene interactions. In order to obtain a comprehensive network for a set of genes, there is a need to integrate several databases, such as PPI, genomic and pathway repositories. CANGES is hypothesis generating tool that provides a significant step forward in obtaining a comprehensive view for a gene set. Furthermore, CANGES is implemented to run on Anduril workflow environment [22], which allows for integration of several protein resources and analysis approaches as exemplified in the glioblastoma multiforme case study.

Many genome-wide SNP arrays, such as Illumina HumanHap arrays, are designed so that the probes query for tag-SNPs instead of central SNPs [23]. The use of tag-SNPs aims at maximizing the amount of variation a SNP captures, which is desirable in many GWA studies. It is, however, challenging to use tag-SNPs to estimate the functional consequences of the observed variation. CANGES compensates the tag-SNP designs by using the HapMap database and producing a comprehensive list of SNPs in region pinpointed by the tag-SNPs. Analysis with functionally designed SNP-arrays such as Affymetrix Drug Metabolism Analysis (DMET) array that contains 1,936 drug metabolism markers in 225 genes or Illumina GoldenGate chip, with approximately 1,500 user-chosen SNPs, target effectively genotypes of several central SNPs that are more amenable to CANGES analysis. Recently several intragenic SNPs have been associated with diseases. These SNPs are usually not the causative variations but surrogate markers indicating the region in which the causative mutation is harbored [24]. Furthermore, the effect of intragenic SNPs to the protein function is currently an unresolved problem. Therefore, CANGES focuses on central SNPs while the intragenic SNPs are discarded from the result lists to avoid dominance of a huge number of the intragenic SNPs.

CANGES provides a means to integrate information from several databases that are accessed through their programming interfaces when such interface is provided. This allows the use of the latest releases of the databases. CANGES may produce very large result sets for queries including genes having a lot of interactions or activity in signaling pathways. In such cases CANGES analysis may take several hours to complete when executed through the CANGES web interface. Therefore, processing of large queries is more efficient through local installations of CANGES than with the website. Furthermore, it is also challenging to estimate the size of the result file because the amount of data in the databases varies for different genes and pathways. The *p53* gene is a prime example of a well-studied gene that produces a very large result set. On the other hand, only three out of six GBM risk genes were found from the pathway- or PPI databases.

Our case studies demonstrate that SNP-protein function prediction tools resulted in widely dissimilar results. For example, in the *p53* case study, only PolyPhen-2 was able to predict that rs1042522 is damaging. Even F-SNP, which uses 16 methods and datasets to predict functional effects of SNP [20], was not able to predict rs1042522 to be damaging (results not shown). The rs1042522 variant causes an amino acid change (R72P) with demonstrated functional consequences; the R72 variant is a stronger and faster inducer of apoptosis than the 72P variant [25], [26] while the 72P variant binds more efficiently to iASPP, an inhibitor of pro-apoptotic function of *p53* [27]. The 72P variant has been found to be more efficient in inducing cell-cycle arrest [25] and DNA repair [28] than the R72 variant. The 72P variant also predicts survival of breast cancer patients [29], [30].

In our second case study we identified genes belonging to the same pathway or directly interacting with protein products of three glioma risk genes and further calculated survival estimates for 1,888 SNPs in these genes. This analysis resulted in a SNP (rs2275565) in the gene Methionine synthase (*MTR*) that encodes the enzyme 5-methyltetrahydrofolate-homocysteine methyltransferase, which catalyzes methione biosynthesis. *MTR* was recently identified as a cancer susceptibility gene regardless of environmental factors [31]. Furthermore, rs2275565 is a tag-SNP for a damaging SNP rs1805087, which was also found in the TCGA dataset showing minor survival effects. Our results therefore suggest that chromosomal region around rs2275565 and rs1805087 is a candidate for harboring a causative variant for the survival effect by *MTR*.

Our results further indicate that also rs100192, which is a tag-SNP for rs164390 in 5′ UTR region of a gene G2/mitotic-specific cyclin-B1 (*CCNB1*), may have an impact on GBM survival. We note that rs164390 itself was not found from the GBM dataset and its effect on the survival could not be estimated directly. This is a common situation in studies integrating data from different cohorts with varying SNP-array designs; and the CANGES results provide a means to rapidly integrate such data. Interestingly, *CCNB1* belongs to the same pathways with *CDKN2A* and *CDKN2B* and is regulated by *p53*. An increase in the copy number in the chromosomal region of *CCNB1* has been shown to be associated with an increase of the cell growth rate in glioma cell lines [32]. Furthermore, upregulation of *CCNB1* along with other cell-cycle genes indicates poor survival in various cancers [33], and upregulation of *CCNB1* alone is suggested to be a marker for a poor prognosis in breast cancer [34], making *CCNB1* an interesting target candidate also in GBM. While it is not surprising to identify to find 18 SNPs with a survival effect from a set of 1,888 SNPs, the two most interesting genes identified here were not be detected in genome-wide context.

**Figure 4:**
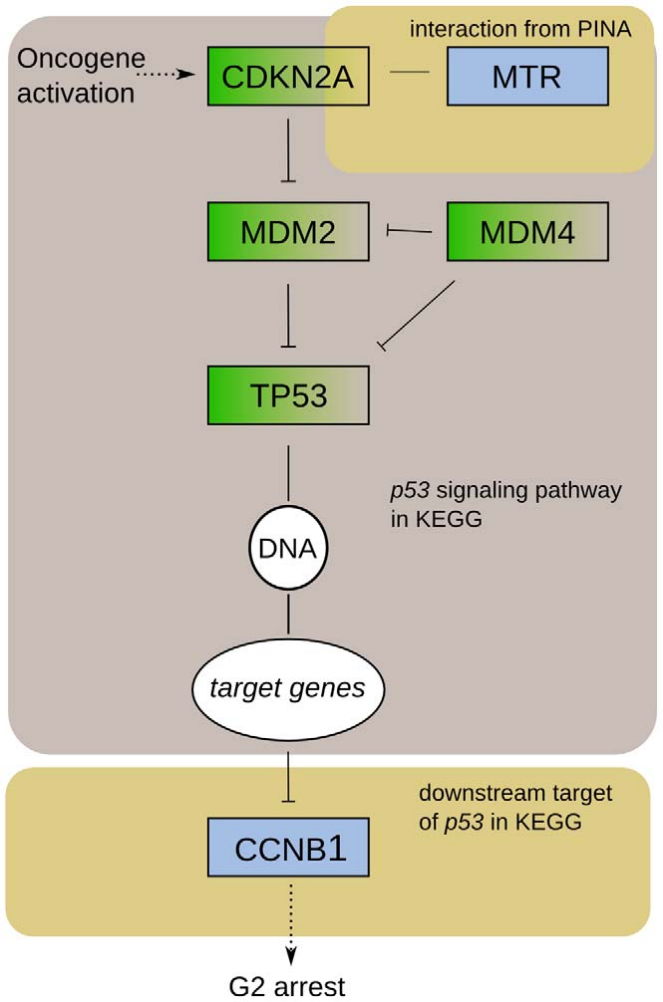
Pathway diagram showing the hypothetical positions of the GBM survival associated genes *MTR* and *CCNB1* in the glioblastoma *p53* pathway [**3**]. KEGG annotates *CCNB1* to belong to the *p53* pathway together with *CCNB2*, *CCNB3* and *CDC2* [6]. In the KEGG *p53* signaling pathway, these are regulated by the genes *SFN*, *GADD45G*, *GADD45A* and *GADD45B*, which constitute a subset of the *p53* target genes. In the KEGG glioma pathway, G2 arrest impairment is annotated as one result of the altered *p53* pathway activation [6].

Both *MTR* and *CCNB1* function in the *p53* pathway, which is altered in 87% of GBM cases [3]. *MTR* interacts with *CDKN2A* [35] and *CCNB1* is a response gene for *p53* [36], [37], which leads to a pathway hypothesis illustrated in Figure 4. Though further studies are required to validate the suggested roles of *MTR* and *CCNB1* in glioblastoma multiforme, our results demonstrate that CANGES is able to produce experimentally testable hypotheses that offer a solid ground for advanced analyses.

## Methods

The CANGES data integration pipeline is divided into three modules, 1) *the protein module*, 2) *the SNP module* and 3) *the evaluation module* as illustrated in Figure 1. Each module can be run individually, which enables four different options for use as described in Table 1.

**Table 1:** Example use cases of CANGES.

CANGES use cases				
	Goal	Input	Databases	Result
1.	Create a set of focal genes or central SNPs based on previously found interesting genes.	List of proteins	KEGG, Reactome, PINA, ENSEMBL, Hapmap, PolyPhen, SNPs3D, SIFT	A list of focal genes and their central SNPs with SNP annotations.
2.	Create a set of focal genes or SNPs based on previously found interesting pathways.	List of pathway names	KEGG, Reactome, PINA, ENSEMBL, Hapmap, PolyPhen, SNPs3D, SIFT	A list of focal genes and their central SNPs with SNP annotations.
3.	Get central SNPs for a custom pathway or a set of interesting genes.	List of genes	ENSEMBL, Hapmap, PolyPhen, SNPs3D, SIFT	A list of central SNPs with annotations.
4.	Find predictions for a set of coding SNPs.	List of genes	PolyPhen, SNPs3D, SIFT	A list of functional predictions for coding SNPs.

The modular structure of CANGES enables selecting inputs, outputs to serve various research goals.

### CANGES workflow

The protein module searches for focal genes from the pathway databases KEGG [6] and Reactome [7], and the PPI database PINA [8], using their programming interfaces. The input for the protein module is a list of proteins or pathways, and the output is a list of focal genes that are either on the search pathway or interacting with at least one of the search proteins through direct interaction or through the same pathway. In the case of the protein list, the protein module searches for all the pathways and PPIs for each protein and creates a list of focal genes from proteins from the resulted pathways and interactions. In the case of the pathway name list, the protein module uses proteins in the query pathways as the list of focal genes.

The SNP module annotates central SNPs for the queried genes from the ENSEMBL genome database [9]. The SNP module excludes all SNPs that are annotated as upstream, downstream, intronic, within non-coding gene or hgmd mutations in ENSEMBL (v. 59), resulting in the central SNPs for the each gene. The SNP module also annotates each SNP with functional, sequence and transcript information as well as information on protein domains in the region of the SNP. A complete list of the annotations in a result file is given in Table S1. We have implemented two redundant interfaces to the ENSEMBL database; the first interface uses BioMart [38] and the second the ENSEMBL Perl. The BioMart interface is much faster than Perl and is thus prioritized. It is, however, also more unstable and the Perl interface allows CANGES to function in cases when BioMart is not responding. The results from the BioMart and Perl interfaces are essentially the same, except that the BioMart implementation collects each SNP from all associated alternative transcripts, while the Perl implementation uses only the first transcript returned by the ENSEMBL database. To avoid time-consuming and redundant searches, the surrounding peptide sequences are fetched only for one alternative transcript if the SNP is located to the same position. The SNP module searches also allele frequencies and tag-SNPs for each annotated SNP, using the HapMap database version 3, draft 1 [10]. The tag-SNPs are selected according to a minimum pair-wise correlation (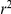) between SNPs in the HapMap database with threshold or as chosen by the user. The allele frequencies and the pair-wise correlations are calculated using the Plink software [39]. The input for the SNP module is a list of protein or gene identifiers, and the output is a list of SNP annotations for each given identifier.

The evaluation module produces predictions for functional effects of SNP to protein function for non-synonymous coding SNPs, using four prediction tools: PolyPhen [11], PolyPhen-2 [12], SNPs3D [13] and SIFT [14]. PolyPhen and PolyPhen-2 produce predictions (*benign*, *possibly damaging*, *probably damaging*) based on the rules regarding the SNPs effect on protein functional sites, the protein structure and the presence of the same peptide substitution in homologous sequences. PolyPhen may produce several predictions for one SNP and all of the predictions are included into the results. SNPs3D calculates scores for SNPs using a protein structure stability model and a feature profile that captures sequence conservation with a support vector machine algorithm. Negative scores predict deleterious effects. The CANGES evaluation module uses the lowest score from the available SNPs3D scores for each SNP. SIFT's predictions are based on the probability of SNP's peptide in homologous sequences, and it considers peptide probability lower than 0.05 damaging for the protein function. SIFT gives a prediction for both SNP's optional peptide variants and the evaluation module selects the lower value indicating more deleterious effect for the prediction. For the predictions, we use local copies of pre-calculated datasets: PolyPhen (54,574 SNPs) and PolyPhen-2 (32,534 SNPs) are from dbSNP build 126 and 130, respectively, whereas SNPs3D uses dbSNP build 128 with 77,999 SNPs and and it uses dbSNP build 129 (178,509 SNPs). The input of the evaluation module is a list of SNP rs-identifiers and the output is the predictions.

### Survival calculations

The effects of GBM focal genes on patient survival were calculated using 209 GBM patient blood samples subjected to 550k SNP-array from the TCGA data set [3]. The survival analyses for all SNPs were conducted with the Kaplan-Meier estimation method and log-rank test, and odds ratios were estimated with logistic regression. In survival analysis we imputed missing values to genotypes having confidence value 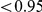, filtered out SNPs with the frequency of missing genotypes 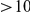
% or minor allele frequency 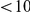 % and set signal to noise ratio 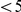 as suggested by [40]. The Kaplan-Meier estimates may become unreliable with small sample sizes. Therefore, we combined the rare homozygous samples with the heterozygous samples when the frequency of homozygotes fell below 10%. This conservative criterion should ensure robust Kaplan-Meier estimates. The combination is optional and the threshold can be assessed by the user. Kaplan-Meier estimates with nominal p-values 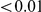 were considered as significant.

### Availability and Future Directions

The CANGES workflow is available as a web service in http://csbi.ltdk.helsinki.fi/CANGES/. The Anduril components are also available at this site. Related databases and the Anduril workflow for the survival analysis are available upon request. Future work focuses on integrating SNP-protein function predictors when such become available. We will also include more variation types from the ENSEMBL database when their consequences on the gene products can be estimated. One future direction of CANGES is to select SNPs for customized high-throughput genotyping experiments.

## Supporting Information

Figure S1 **The Kaplan–Meier curves from the 16 SNPs showing survival effect from the glioblastoma multiforme case study.**
(PDF)

Table S1 **The complete description of annotations from both ENSEMBL Perl and BioMart interfaces.**
(XLS)

Table S2 **The list of proteins and their SNP annotations (ENSEMBL Perl interface) of focal genes from the** ***p53*** **case study.**
(XLS)

Table S3 **The list** ***p53*** **focal genes' coding SNPs that all prediction methods in CANGES estimate of being functional.**
(XLS)

Table S4 **The list of the central SNPs and their annotations (ENSEMBL Perl interface) from the glioblastoma multiforme case study.**
(XLS)

Table S5 **The central SNPs from the glioblastoma multiforme case study that show a survival effect either itself or through tagSNPs.** Columns from survival analysis are the following: *SurvivalMarker  = * the SNP for which survival is estimated, *Hetero OR  = * the odds ratio between heterozygous and homozygous genotypes, *Homo OR  = * the odds ratio between homozygous genotypes, *pvalue*  =  the p-value.
(XLS)
