# Supplementary material for: Data Integration Workflow for Search of Disease Driving Genes and Genetic Variants
Source: PLoS One. 2011 Apr 12;6(4):e18636. doi: 10.1371/journal.pone.0018636 (PMC3075259; doi:10.1371/journal.pone.0018636)

Survival analysis of rs607230

This marker has a genotype specific effect with a p-value of 5.356426e-03.

Odds ratios have been estimated using logistic regression at $t \approx 36$:

$$\frac{P(event|AT,AA)/P(\neg event|AT,AA)}{P(event|TT)/P(\neg event|TT)} \approx 0.354.$$

genotype	total N	events	censored
TT	115	96 (0.835)	19 (0.165)
AT,AA	92	59 (0.641)	33 (0.359)

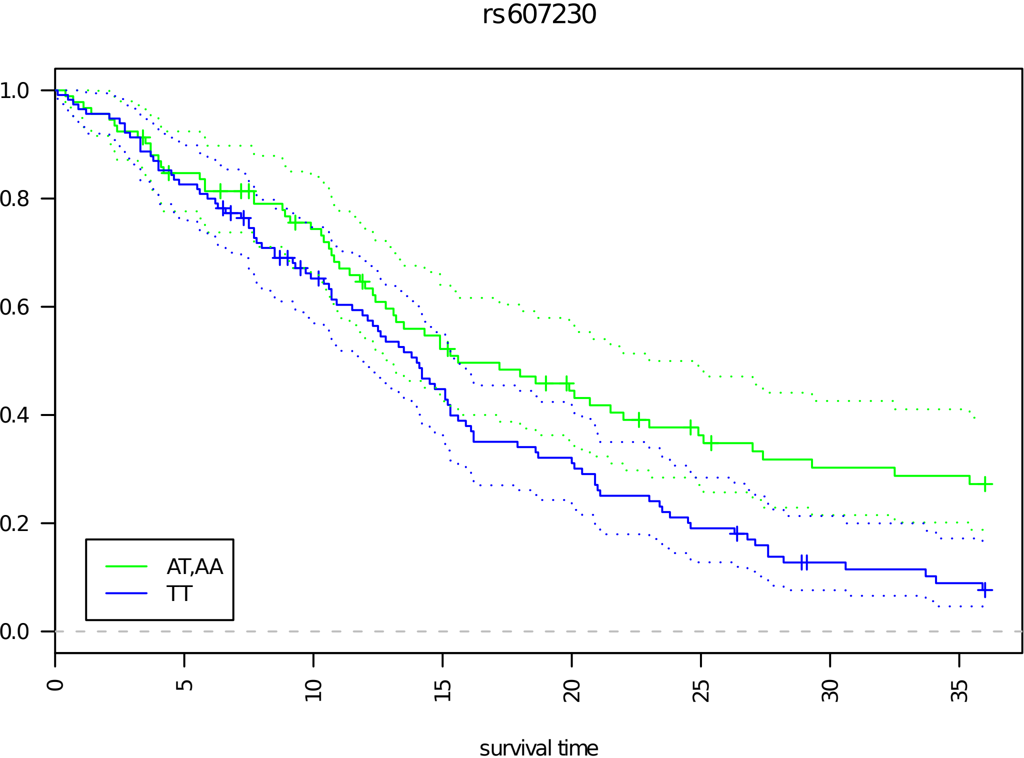

Survival analysis of rs17532679

This marker has a genotype specific effect with a p-value of 3.726115e-03.

Odds ratios have been estimated using logistic regression at $t \approx 36$:

$$\frac{P(\text{event}|AT,TT)/P(\neg\text{event}|AT,TT)}{P(\text{event}|AA)/P(\neg\text{event}|AA)} \approx 2.692.$$

genotype	total N	events	censored
AA	131	90 (0.687)	41 (0.313)
AT,TT	76	65 (0.855)	11 (0.145)

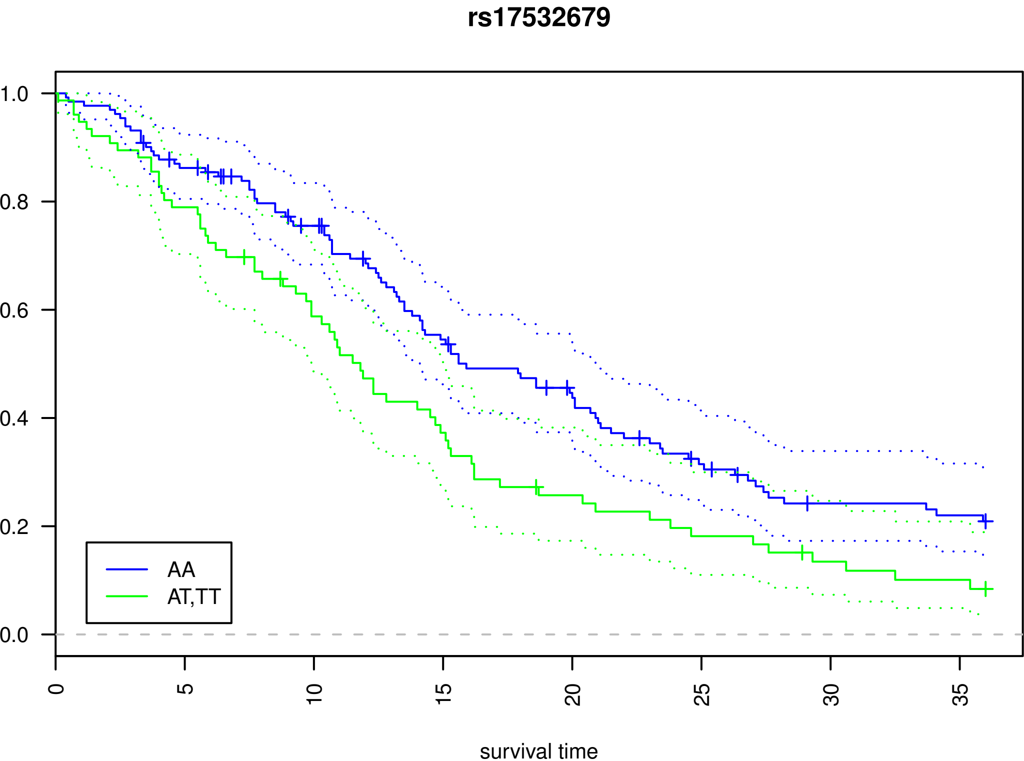

Survival analysis of rs154238

This marker has a genotype specific effect with a p-value of 9.466737e-03. Total of 1 observation deleted due to missingness. Odds ratios have been estimated using logistic regression at $t \approx 36$:

$$\frac{P(\text{event}|AT,TT)/P(\neg\text{event}|AT,TT)}{P(\text{event}|AA)/P(\neg\text{event}|AA)} \approx 1.826.$$

.

genotype	total N	events	censored
AA	121	85 (0.702)	36 (0.298)
AT,TT	85	69 (0.812)	16 (0.188)

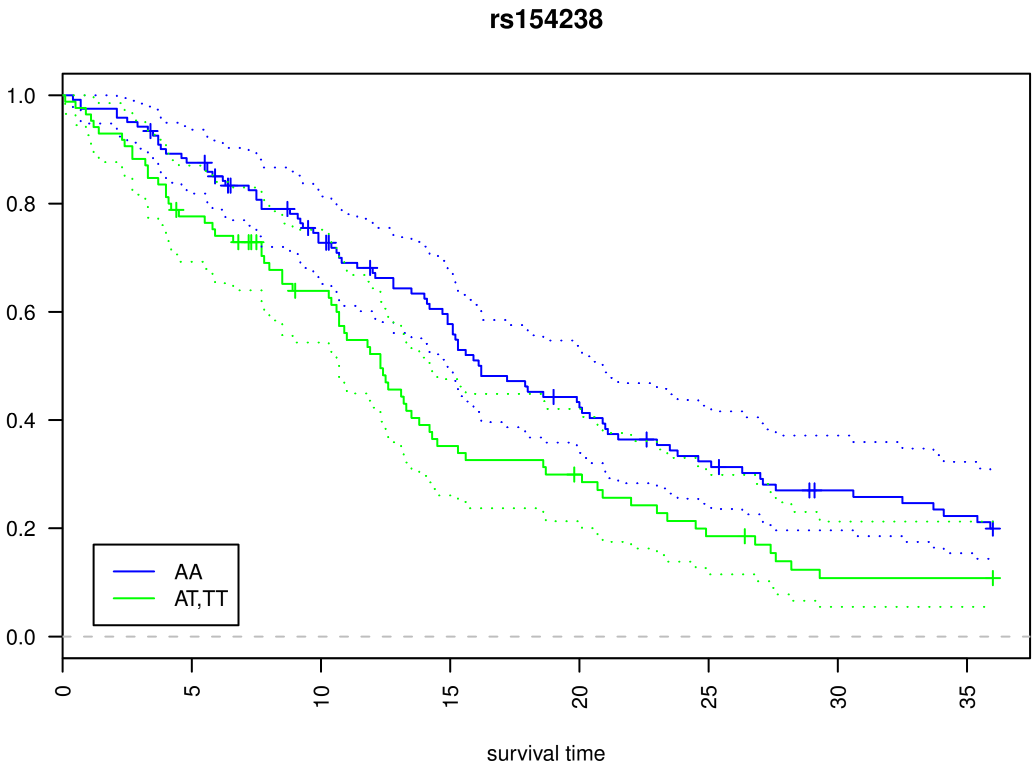

Survival analysis of rs4561519

This marker has a genotype specific effect with a p-value of 7.774728e-03.

Odds ratios have been estimated using logistic regression at $t \approx 36$:

$$\frac{P(\text{event}|AT,AA)/P(\neg\text{event}|AT,AA)}{P(\text{event}|TT)/P(\neg\text{event}|TT)} \approx 2.779.$$

genotype	total N	events	censored
TT	141	98 (0.695)	43 (0.305)
AT,AA	66	57 (0.864)	9 (0.136)

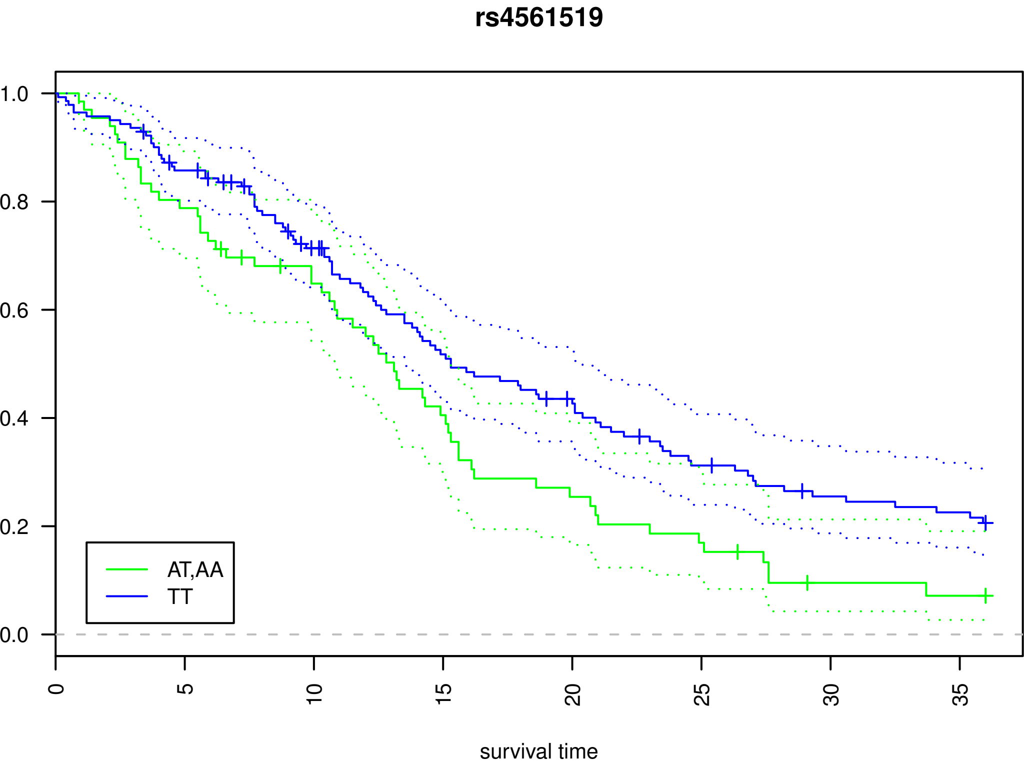

Survival analysis of rs7236987

This marker has a genotype specific effect with a p-value of 7.135140e-03.

Odds ratios have been estimated using logistic regression at $t \approx 36$:

$$\frac{P(\text{event}|AT,TT)/P(\neg\text{event}|AT,TT)}{P(\text{event}|AA)/P(\neg\text{event}|AA)} \approx 0.369.$$

genotype	total N	events	censored
AA	155	124 (0.8)	31 (0.2)
AT,TT	52	31 (0.596)	21 (0.404)

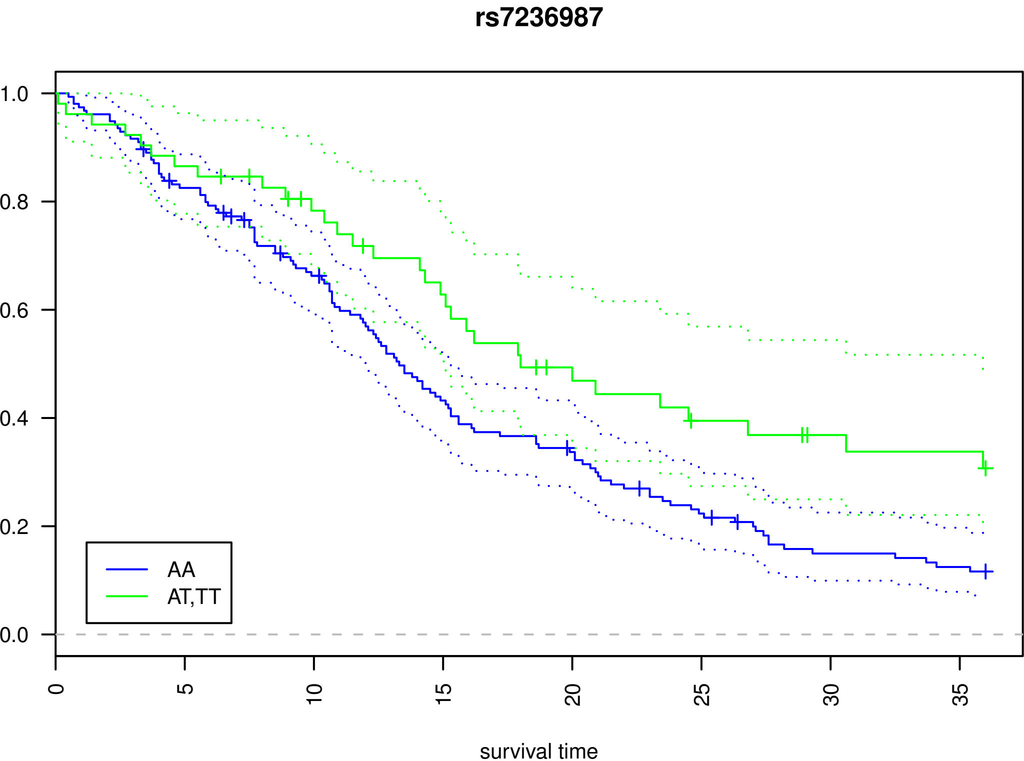

Survival analysis of rs3809908

This marker has a genotype specific effect with a p-value of 5.311930e-03. Total of 1 observation deleted due to missingness. Odds ratios have been estimated using logistic regression at $t \approx 36$:

$$\frac{P(\text{event}|AT,TT)/P(\neg\text{event}|AT,TT)}{P(\text{event}|AA)/P(\neg\text{event}|AA)} \approx 0.357.$$

genotype	total N	events	censored
AA	154	124 (0.805)	30 (0.195)
AT,TT	52	31 (0.596)	21 (0.404)

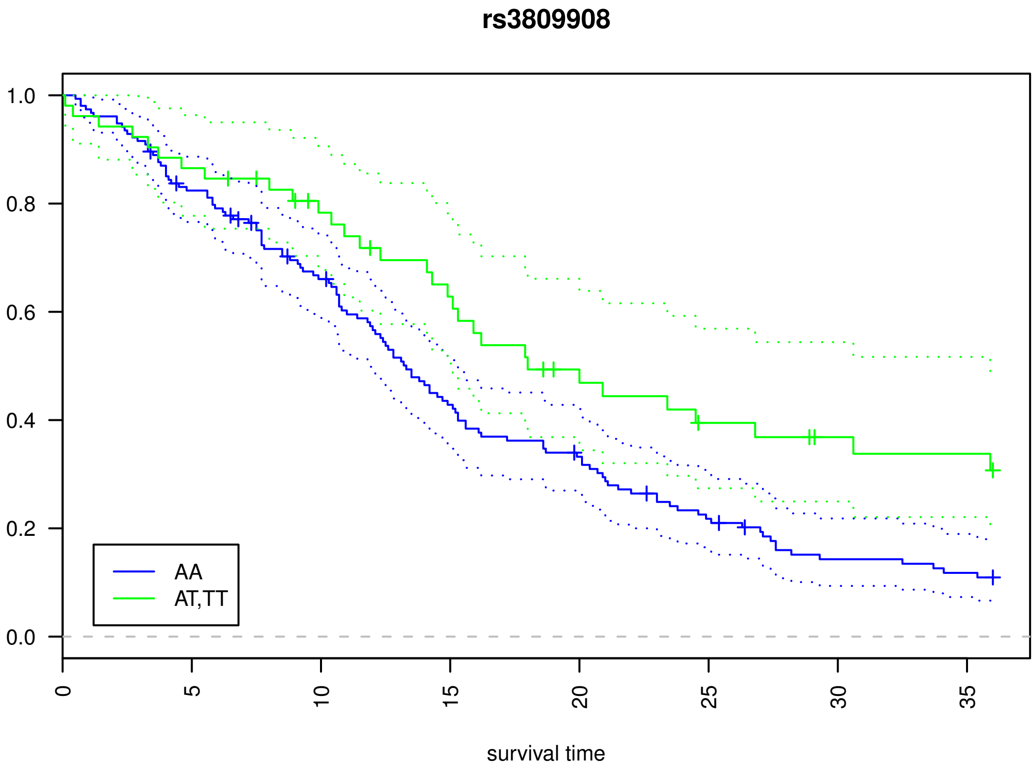

Survival analysis of rs6077759

This marker has a genotype specific effect with a p-value of 3.812266e-03.

Odds ratios have been estimated using logistic regression at $t \approx 36$:

$$\frac{P(\text{event}|AT)/P(\neg\text{event}|AT)}{P(\text{event}|AA)/P(\neg\text{event}|AA)} \approx 1.634 \text{ and } \frac{P(\text{event}|TT)/P(\neg\text{event}|TT)}{P(\text{event}|AA)/P(\neg\text{event}|AA)} \approx 2.93.$$

genotype	total N	events	censored
AA	103	71 (0.689)	32 (0.311)
AT	74	58 (0.784)	16 (0.216)
TT	30	26 (0.867)	4 (0.133)

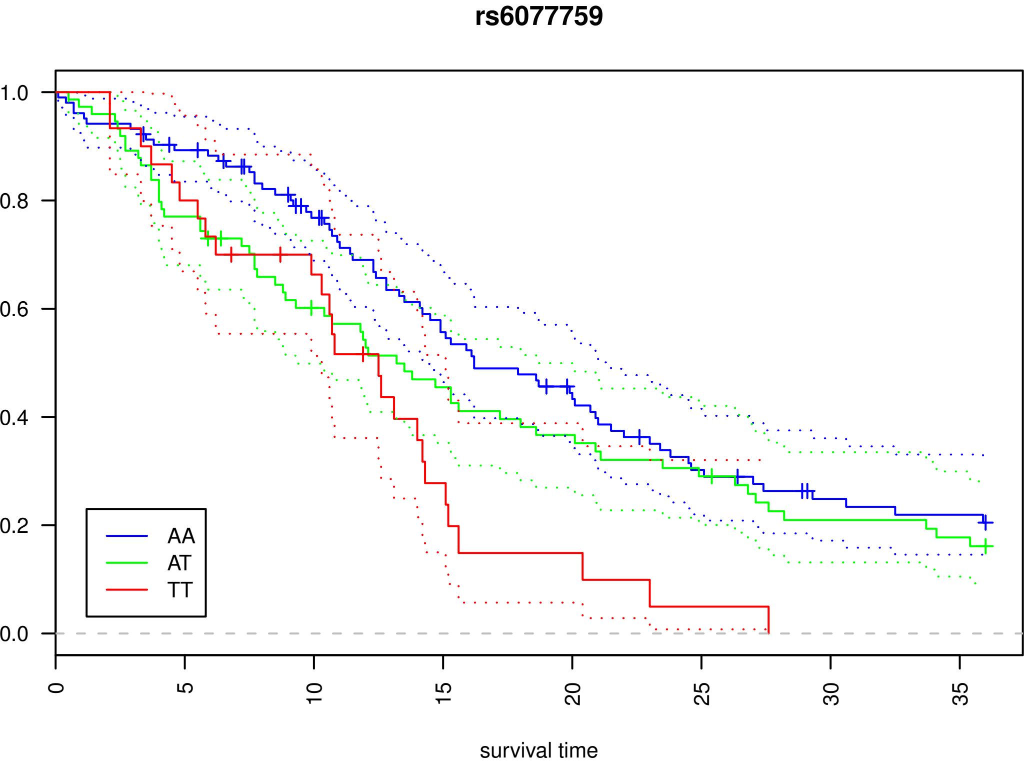

Survival analysis of rs6772

This marker has a genotype specific effect with a p-value of 7.746774e-03.

Odds ratios have been estimated using logistic regression at t ≈ 36:

$$\frac{P(\text{event}|AT,AA)/P(\neg\text{event}|AT,AA)}{P(\text{event}|TT)/P(\neg\text{event}|TT)} \approx 0.412.$$

genotype	total N	events	censored
TT	125	102 (0.816)	23 (0.184)
AT,AA	82	53 (0.646)	29 (0.354)

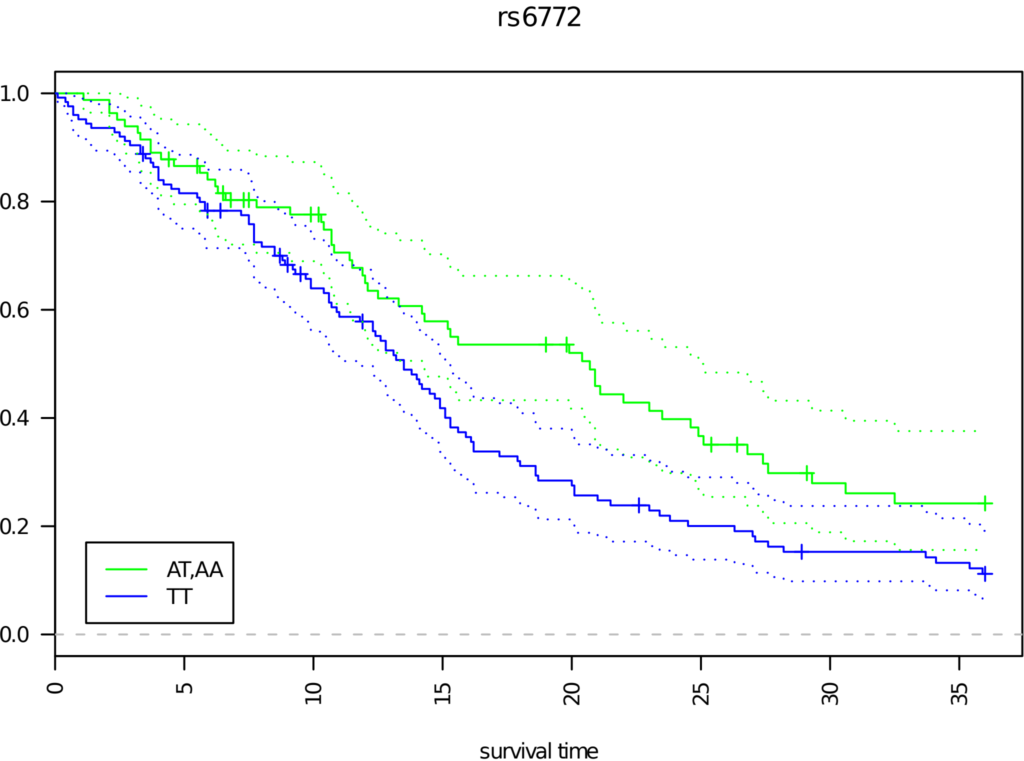

Survival analysis of rs11242316

This marker has a genotype specific effect with a p-value of 7.062309e-03.

Odds ratios have been estimated using logistic regression at t ≈ 36:

$$\frac{P(event|AT)/P(\neg event|AT)}{P(event|TT)/P(\neg event|TT)} \approx 2.047 \text{ and } \frac{P(event|AA)/P(\neg event|AA)}{P(event|TT)/P(\neg event|TT)} \approx 0.746.$$

genotype	total N	events	censored
TT	113	81 (0.717)	32 (0.283)
AT	68	57 (0.838)	11 (0.162)
AA	26	17 (0.654)	9 (0.346)

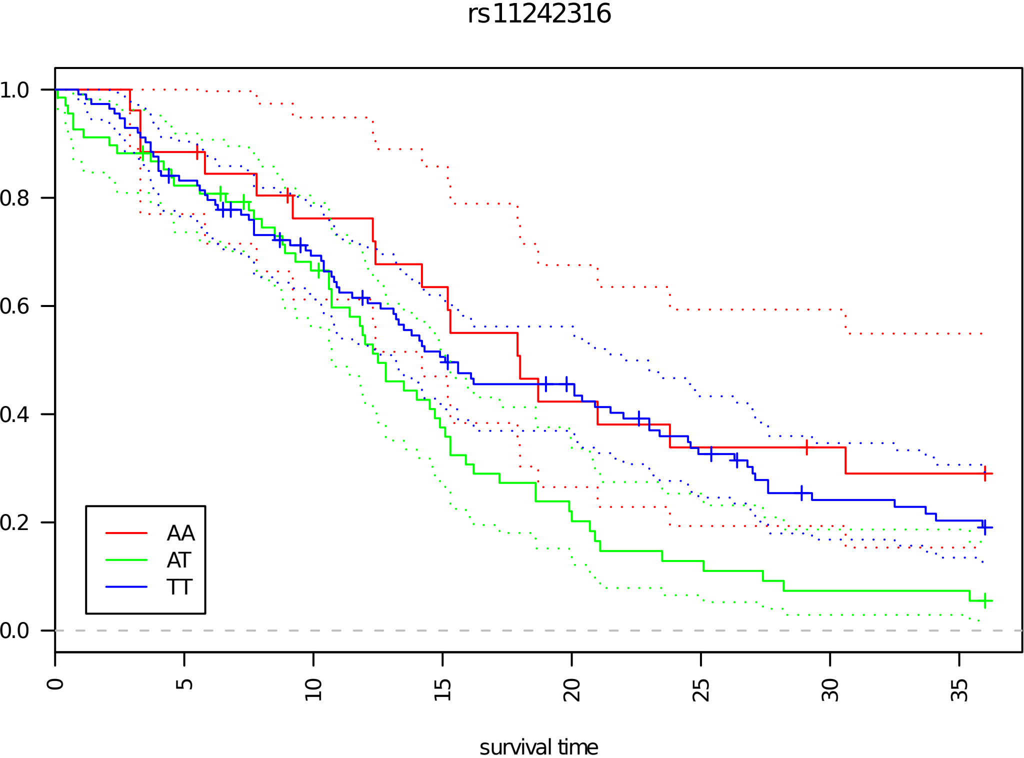

Survival analysis of rs10158222

This marker has a genotype specific effect with a p-value of 4.042413e-03.

Odds ratios have been estimated using logistic regression at t ≈ 36:

$$\frac{P(\text{event}|AT,TT)/P(\neg\text{event}|AT,TT)}{P(\text{event}|AA)/P(\neg\text{event}|AA)} \approx 0.632.$$

genotype	total N	events	censored
AA	121	95 (0.785)	26 (0.215)
AT,TT	86	60 (0.698)	26 (0.302)

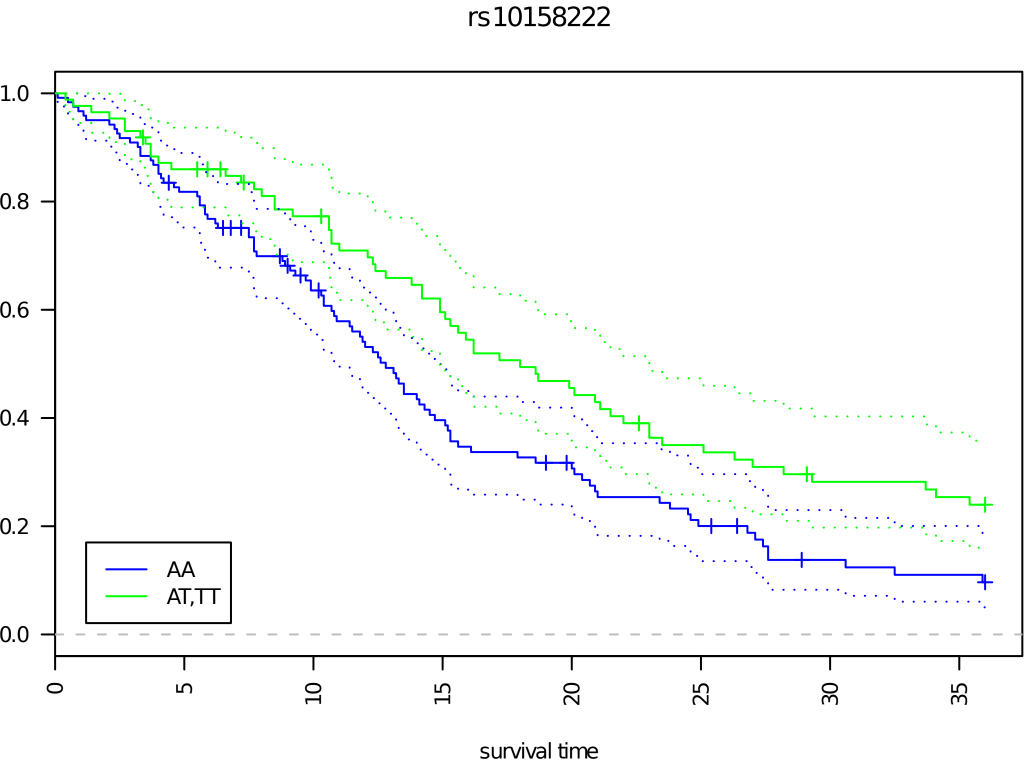

Survival analysis of rs10045073

This marker has a genotype specific effect with a p-value of 9.680628e-03.

Odds ratios have been estimated using logistic regression at $t \approx 36$:

$$\frac{P(\text{event}|AT)/P(\neg\text{event}|AT)}{P(\text{event}|TT)/P(\neg\text{event}|TT)} \approx 1.963 \text{ and } \frac{P(\text{event}|AA)/P(\neg\text{event}|AA)}{P(\text{event}|TT)/P(\neg\text{event}|TT)} \approx 0.685.$$

.

genotype	total N	events	censored
TT	115	83 (0.722)	32 (0.278)
AT	67	56 (0.836)	11 (0.164)
AA	25	16 (0.64)	9 (0.36)

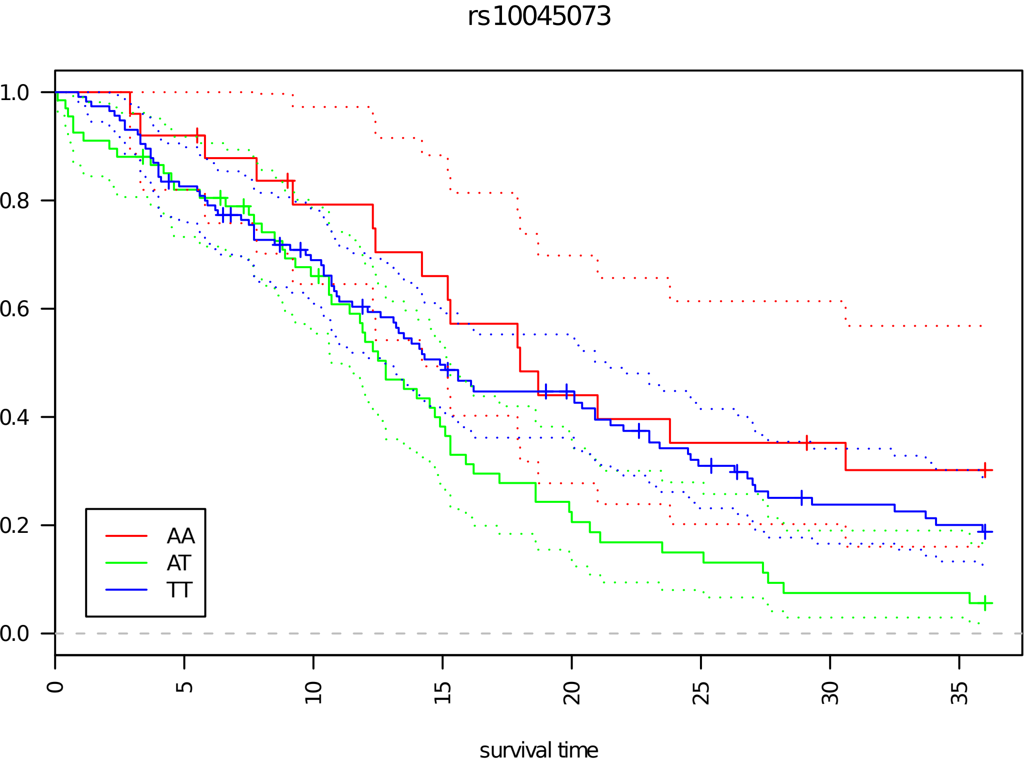

Survival analysis of rs7107335

This marker has a genotype specific effect with a p-value of 4.849804e-03. Total of 1 observation deleted due to missingness. Odds ratios have been estimated using logistic regression at t ≈ 36:

$$\frac{P(\text{event}|AT,AA)/P(\neg\text{event}|AT,AA)}{P(\text{event}|TT)/P(\neg\text{event}|TT)} \approx 0.554.$$

genotype	total N	events	censored
TT	136	107 (0.787)	29 (0.213)
AT,AA	70	47 (0.671)	23 (0.329)

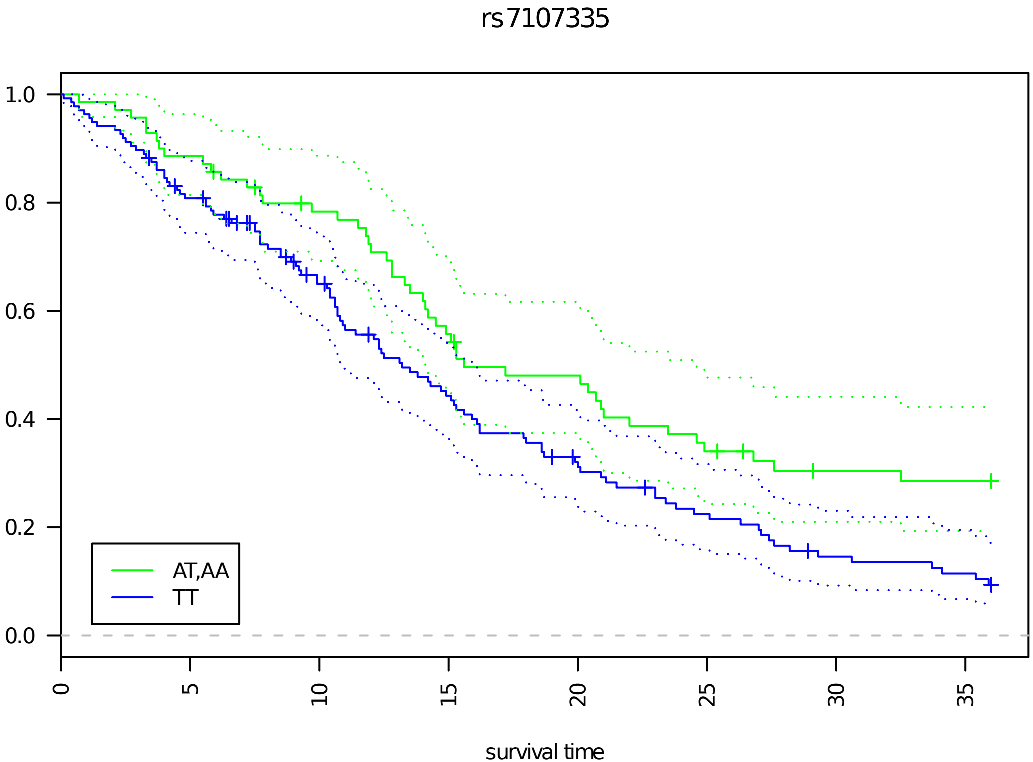

Survival analysis of rs6886699

This marker has a genotype specific effect with a p-value of 9.597962e-03.

Odds ratios have been estimated using logistic regression at t ≈ 36:

$$\frac{P(\text{event}|AT)/P(\neg\text{event}|AT)}{P(\text{event}|TT)/P(\neg\text{event}|TT)} \approx 2.085 \text{ and } \frac{P(\text{event}|AA)/P(\neg\text{event}|AA)}{P(\text{event}|TT)/P(\neg\text{event}|TT)} \approx 0.805.$$

.

genotype	total N	events	censored
TT	115	82 (0.713)	33 (0.287)
AT	68	57 (0.838)	11 (0.162)
AA	24	16 (0.667)	8 (0.333)

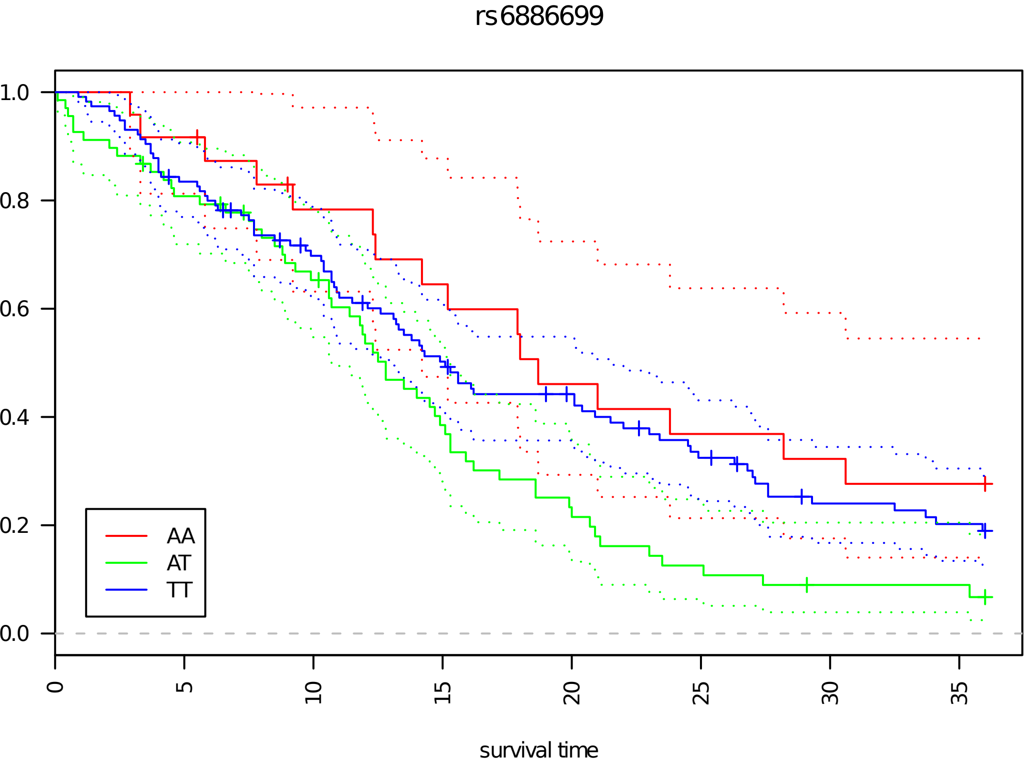

Survival analysis of rs3734125

This marker has a genotype specific effect with a p-value of 2.445427e-03.

Odds ratios have been estimated using logistic regression at t ≈ 36:

$$\frac{P(\text{event}|AT)/P(\neg\text{event}|AT)}{P(\text{event}|TT)/P(\neg\text{event}|TT)} \approx 2.083 \text{ and } \frac{P(\text{event}|AA)/P(\neg\text{event}|AA)}{P(\text{event}|TT)/P(\neg\text{event}|TT)} \approx 0.702.$$

genotype	total N	events	censored
TT	113	81 (0.717)	32 (0.283)
AT	69	58 (0.841)	11 (0.159)
AA	25	16 (0.64)	9 (0.36)

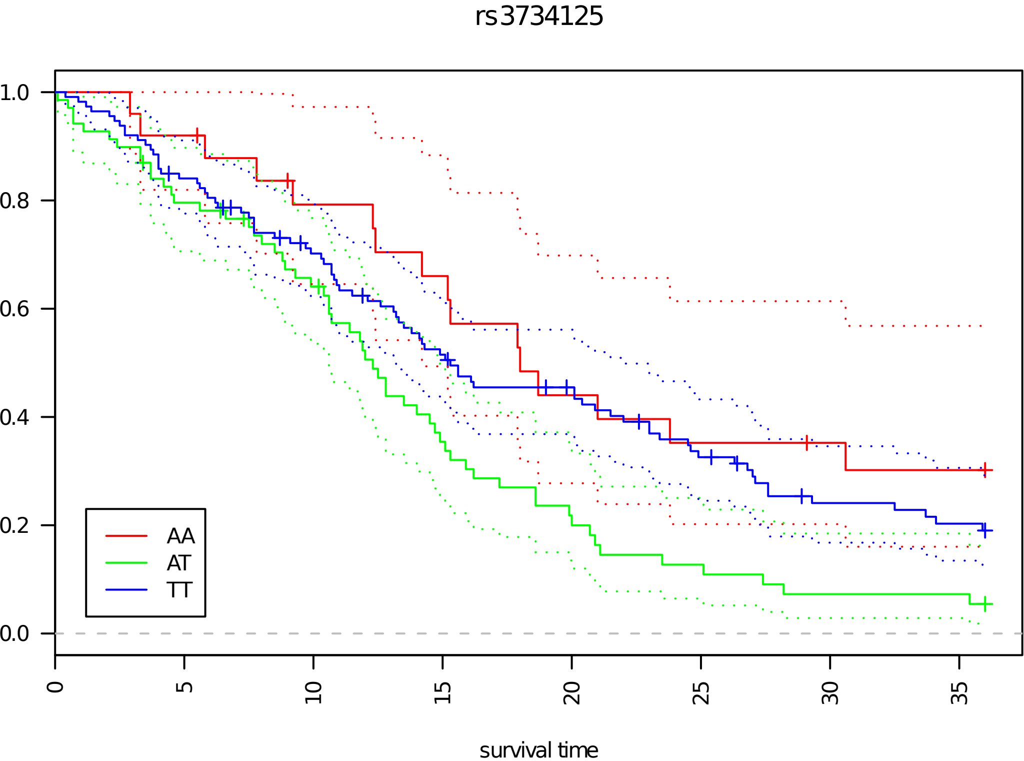

Survival analysis of rs3733619

This marker has a genotype specific effect with a p-value of 6.766583e-03.

Odds ratios have been estimated using logistic regression at t ≈ 36:

$$\frac{P(event|AT,AA)/P(\neg event|AT,AA)}{P(event|TT)/P(\neg event|TT)} \approx 5.681.$$

genotype	total N	events	censored
TT	164	115 (0.701)	49 (0.299)
AT,AA	43	40 (0.93)	3 (0.0698)

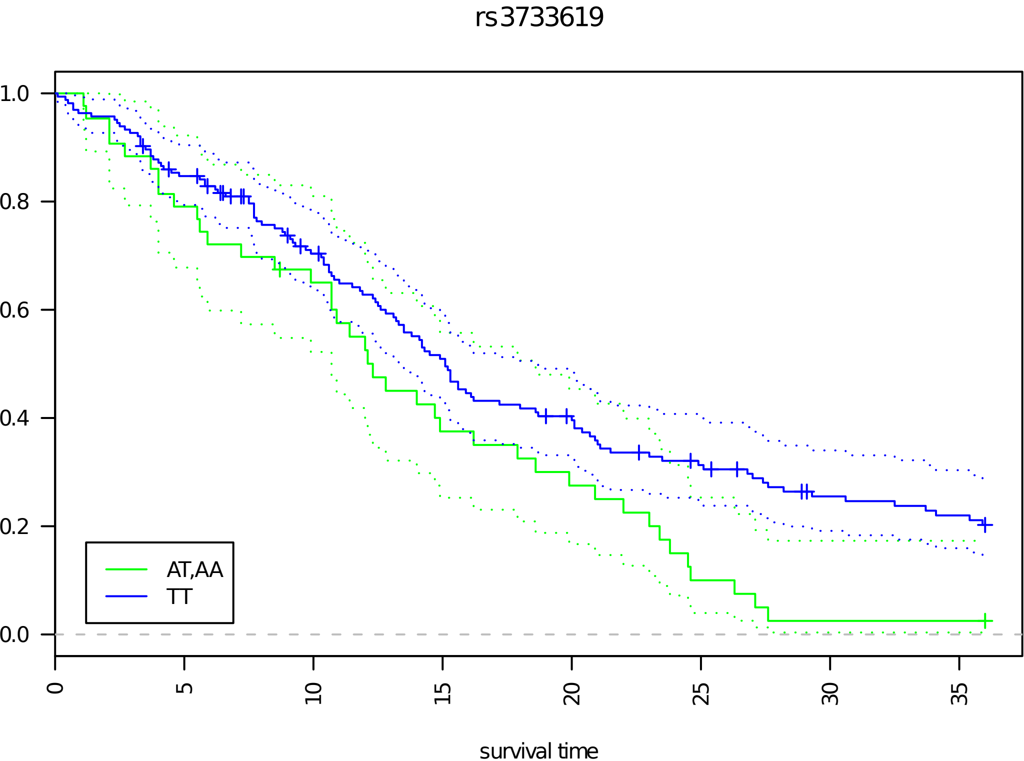

Survival analysis of rs2491237

This marker has a genotype specific effect with a p-value of 5.246671e-03.

Odds ratios have been estimated using logistic regression at t ≈ 36:

$$\frac{P(\text{event}|AT,AA)/P(\neg\text{event}|AT,AA)}{P(\text{event}|TT)/P(\neg\text{event}|TT)} \approx 0.412.$$

genotype	total N	events	censored
TT	125	102 (0.816)	23 (0.184)
AT,AA	82	53 (0.646)	29 (0.354)

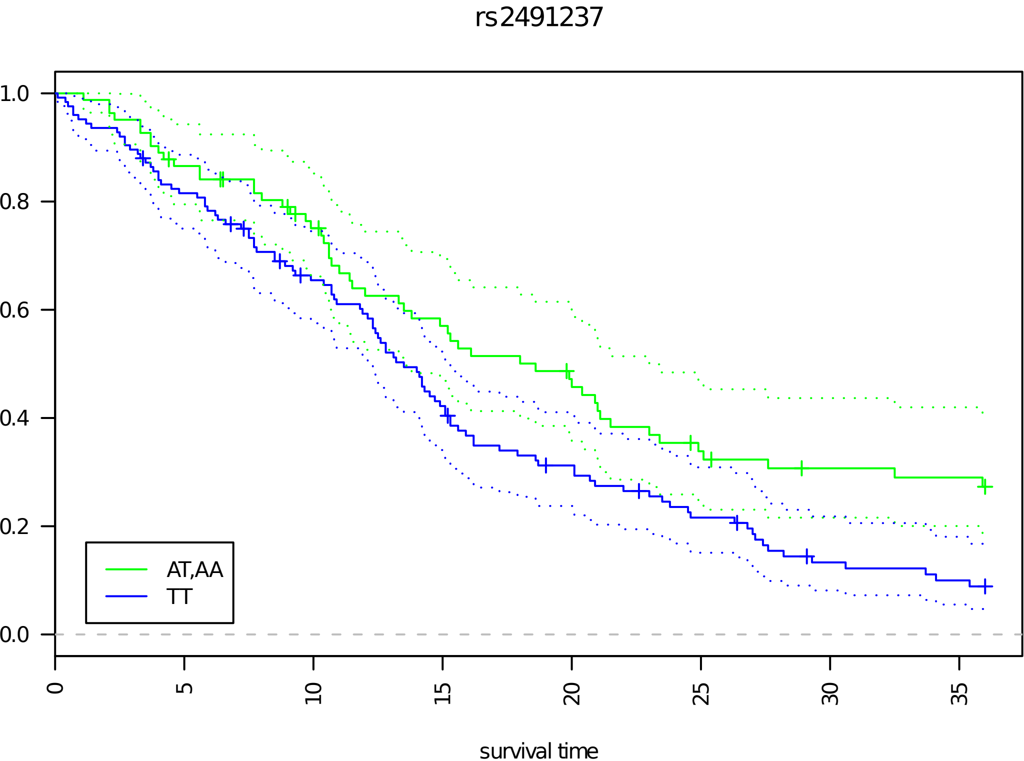

Supplement: Figure S1 — The Kaplan–Meier curves from the 16 SNPs showing survival effect from the glioblastoma multiforme case study. (PDF) [file pone.0018636.s001.pdf]
